# Supplementary material for: A quantitative hypermorphic CNGC allele confers ectopic calcium flux and impairs cellular development
Source: eLife. 2017 Sep 21;6:e25012. doi: 10.7554/eLife.25012 (PMC5716663; doi:10.7554/eLife.25012)
Supplement: Supplementary file 1. [file elife-25012-supp1.pdf]

Supplementary File 1. Oligonucleotides used in this study.

Oligo ID	Sequence 5'-3'	Purpose
SNP12 F	CATTGAATATATAAAACAACAACC	SNP Amplification
SNP12 R	GGAAGGTTTGGATGCGAATA	SNP Amplification
SNP24-1 F	CCCGAGAAAAATGCAGCTTA	SNP Amplification
SNP24-1 R	CGGCCAGTACCAAGCTAAAG	SNP Amplification
SNP43 F	TCCGTCATGAACATTGAGATG	SNP Amplification
SNP43 R	CTCCTAAAGATAGCGTTGAGC	SNP Amplification
SNP3 F	AGATTGTCTTGCGCGTATGT	SNP Amplification
SNP3 R	ACAATGCGGATTCTGAGGTC	SNP Amplification
KH142 F	TCGTTTGGCCTTTCATTCTG	Sequencing *BRUSH*
KH143 R	AATGTAAAGGGAGGCCAGAG	Sequencing *BRUSH*
KH144 F	CCAAGTGGTATTCCCCATCA	Sequencing *BRUSH*
KH145 R	TGTGCAAAATTACTACTTAAGGAAC	Sequencing *BRUSH*
KH146 F	AAGGTACTCATCAAAATACTTGTCA	Sequencing *BRUSH*
KH147 F	TCACTAAAATTCCCACAAATGGT	Sequencing *BRUSH*
KH148 R	GGGATAGGGAATTCATCAGGA	Sequencing *BRUSH*
KH149 F	TCACTTTATTTAGGTTCTATGCTACA	Sequencing *BRUSH*
KH150 R	CACTTTACAATCTAGTTCTCTATGG	Sequencing *BRUSH*
KH219 F	GTAAAACGACGGCCAGTCTGCAGGAGCTGGAAATCAT	dCAPS *BRUSH*
KH180 R	AGGATCATTACACATTCCCAGCGGC	dCAPS *BRUSH*
DC1 F	ATGGTGTCTGGTTCGCAGTA	*ELF1* qPCR
DC2 R	ATAGTCTTGCCACCGTCGAT	*ELF1* qPCR
DC7 F	CCAAGGAGCTCTAAGGTATGAA	*BRUSH* qPCR
DC8 R	TACGACTTCGGTGTTTGATCTG	*BRUSH* qPCR
DC9 F	CTTCCAGGACAAAGGTTGCTT	*CNGC.IVA2* qPCR
DC10 R	GAGGTCCTTGAAAACGTAGAC	*CNGC.IVA2* qPCR
DC11 F	CCTTACTGGAGATCCCTTGC	*CNGC.IVA3* qPCR
DC12 R	TCAAAGCCCTTGTGAGGTATTAG	*CNGC.IVA3* qPCR
DC13 F	GACAAGGGTTGCTCAGCAGT	*CNGC.IVA4* qPCR
DC14 R	AACACGAGGACTGTGCAAGAA	*CNGC.IVA4* qPCR
DC15 F	GCTTTAAGGTATGAATCGCCTTAT	*CNGC.IVA5* qPCR
DC16 R	CCTCAATGTTTAGTTGTTACTGAG	*CNGC.IVA5* qPCR
KH172 F	CACCATGCCTCAATTCGACAAAG	*BRUSH* *g*CDS pENTR
KH169 R	CGACTTCGGTGTTTGATCTG	*BRUSH* *g*CDS pENTR
KH182 F	CACCGTCGACTCGTGTTGTCACTCCATTCA	*BRUSH* PRO pK7RWG2
KH183 R	ACTGTCGACGTTTCCTTGACGTTTCTGTAGT	*BRUSH* PRO pK7RWG2
DC17 1F	ATGGTCTCACACCATGCCTCAATTCGACAAAGA	*BRUSH* CDS pENTR-Bsa
DC18 1R	TAGGTCTCTGTCCTTCTGGCAAGCGAC	*BRUSH* CDS pENTR-Bsa
DC19 2F	TAGGTCTCTGGACTCAGAAGGAAAGTACGTCA	*BRUSH* CDS pENTR-Bsa
DC20 2R	ATGGTCTCACCTTCGACTTCGGTGTTTGATCTG	*BRUSH* CDS pENTR-Bsa
DC77 1F	TTGAAGACATTACGGGTCTCACACCATGCCTCAATTCGACAAAGA	*BRUSH* CDS LI GG
DC58 R1	TAGGTCTCTTTTTCTGTTTAAGTCTCTCACGAAT	*BRUSH* CDS LI GG
DC59 2F	TAGGTCTCTAAAACATACATCAAAGGAAGTAGAA	*BRUSH* CDS LI GG

DC78 1R	ATGAAGACTTCAGAGGTCTCACCTTCGACTTCGGTGTTTGATCTG	*BRUSH* CDS LI GG
DC824 R	ATGAAGACTTCAGAGGTCTCTCCTTTTGTTGTACAACTTTAGCGTGAGG	*BRUSH* NT pENTR-Bsa
DC370	ATGGTCTCACACCATGCATCAGGCTCACACTTCAT	*BRUSH* 5’RNAi LI GG
DC371	TAGGTCTCTCCTTGTCGAATTGAGGCATGTTTCCTT	*BRUSH* 5’RNAi LI GG
DC372	ATGGTCTCACACCGGTCGTGCTCGTGCTAATATCT	*BRUSH* 3’RNAi LI GG
DC373	TAGGTCTCTCCTTGGCAGAAAATAAGTTGTTTTATGATAATG	*BRUSH* 3’RNAi LI GG
DC673 F1	ATGGTCTCACACCATGGCTCAATTCGATAAAGATGAGG	*CNGC.IVA3* CDS pENTR-Bsa
DC674 R1	TAGGTCTCTGAGCCCATAGGAATAGACATTGATG	*CNGC.IVA3* CDS pENTR-Bsa
DC675 F2	ATGGTCTCAGCTCCATGGAACCATATGATAC	*CNGC.IVA3* CDS pENTR-Bsa
DC676 R2	TAGGTCTCTCCTTAAGCCCTTGTGAGGTATTAGCAC	*CNGC.IVA3* CDS pENTR-Bsa
DC1028 R	ATGAAGACTTCAGAGGTCTCTCCTTATGTTGTACAACTTTAGCGTGCGG	*CNGC.IVA3* NT pENTR-Bsa
DC677 F	ATGGTCTCACACCCACCATGGCCAGTTCTGAAAACAATGAG	*CNGC.IVA4* CDS pENTR-Bsa
DC678 R	TAGGTCTCTCCTTACCTGATAATGACGAATGAGCGC	*CNGC.IVA4* CDS pENTR-Bsa
DC1029 R	ATGAAGACTTCAGAGGTCTCTCCTTTTGTTGTATGAACTTAGTGTGAGG	*CNGC.IVA4* NT pENTR-Bsa
DC679 F	ATGGTCTCACACCCACCATGGCTAATTTTAAAAATGATGAGGTGC	*CNGC.IVA4* CDS pENTR-Bsa
DC680 R	TAGGTCTCTCCTTATGTTTAGTTGTTACTGAGGAATTAACA	*CNGC.IVA5* CDS pENTR-Bsa
DC1030 R	ATGAAGACTTCAGAGGTCTCTCCTTTTGTTGAATGACTTTAGTGTGAGG	*CNGC.IVA5* NT pENTR-Bsa
DC60 1F	TTGAAGACATTCAGGGTCTCAGCGGTCGTGTTGTCACTCCATTCAAAAA	*BRUSH* Pro LI GG
DC61 1R	TAGAAGACTGCTCTCTTAAAAGTATATCTACCCTG	*BRUSH* Pro LI GG
DC62 2F	TAGAAGACTGAGCATAACCACTAAAACCATAGCTC	*BRUSH* Pro LI GG
DC63 2R	TAGAAGACTCACAAGTTTTAATGGAAGAGTTAACAA	*BRUSH* Pro LI GG
DC64 3F	ATGAAGACATGTGACCTTTGATAGTATTTAAATTACAC	*BRUSH* Pro LI GG
DC65 3R	TTGAAGACATCAGAGGTCTCACAGAGTTTCCTTGACGTTTCTGTAGTTTT	*BRUSH* Pro LI GG
DC646 F	ATGAAGACTTTACGGGTCTCAgcggTAATACGACTCACTATAGGGCG	T7 β-Globin A-B
DC647 R	ATGAAGACTTCAGAGGTCTCTcagaGGCCAAAGTTGAGCGTTTATTC	T7 β-Globin A-B
DC648 F	ATGAAGACTTTACGGGTCTCAaatcACCAGCCTCAAGAACACCC	3’UTR β-Globin E-F
DC649 R	ATGAAGACTTCAGAGGTCTCTctcaTGTGAAGAAACTTTCTTTTTATTAGGA	3’UTR β-Globin E-F
DC916 F	ATGAAGACTTTACGGGTCTCATCTGaacaATGGTGAGCAAGGGCGAGGA	VYNE(R) B-C BiFC
DC917 R	ATGAAGACTTCAGAGGTCTCAGGTGccAAGATCCTCCTCAGAAATCAACTT	VYNE(R) B-C BiFC
DC918 F	ATGAAGACTTTACGGGTCTCATCTGaacaATGGACAAGCAGAAGAACGGC	mVYCE(R) B-C BiFC
DC919 R	ATGAAGACTTCAGAGGTCTCAGGTGccAGCGTAATCTGGAACATCGTATG	mVYCE(R) B-C BiFC
DC920 F	ATGAAGACTTTACGGGTCTCAAAGGgaATGGAGCAAAAGTTGATTTCTGAG	VYNE D-E BiFC
DC921 R	ATGAAGACTTCAGAGGTCTCAGATTCTACTCGATGTTGTGGCGGAT	VYNE D-E BiFC
DC922 F	ATGAAGACTTTACGGGTCTCAAAGGgaATGTACCCATACGATGTTCCAG	mVYCE D-E BiFC
DC923 R	ATGAAGACTTCAGAGGTCTCAGATTTTACTTGTACAGCTCGTCCATG	mVYCE D-E BiFC
DC984 F1	ATGAAGACTTTACGGGTCTCAGCGGATCACATCAGCCTCCCTATCC	*CNGC.IVA3* Pro LI GG
DC985 R1	TAGAAGACAAGTCACTCTTGTATTGAACCGCAAG	*CNGC.IVA3* Pro LI GG
DC986 F2	TAGAAGACAATGACCCTCATGGCTTCACGCAA	*CNGC.IVA3* Pro LI GG
DC987 R2	TAGAAGACAAAAGTCTTTGACAAAGTCATTTGTTGAG	*CNGC.IVA3* Pro LI GG
DC988 F3	TAGAAGACAAACTTCCTTCATCTTCACAAGTAC	*CNGC.IVA3* Pro LI GG
DC989 R3	ATGAAGACTTCAGAGGTCTCACAGAGTTTCCTTCACGATTCTGCAGT	*CNGC.IVA3* Pro LI GG
DC1031 F1	ATGAAGACTTTACGGGTCTCAGCGG CGC CTT GTC CAT CAA CTC TCT T	*CNGC.IVA4* Pro LI GG
DC979 R2	TAGAAGACAAATGTAGACCTCAACTTTTCAGGACT	*CNGC.IVA4* Pro LI GG
DC980 F3	TAGAAGACAAACATCTGGAACAAATCTAAATTGCA	*CNGC.IVA4* Pro LI GG
DC981 R3	ATGAAGACTTCAGAGGTCTCACAGAGCTTCCTTGACAATTCTATAATTTT	*CNGC.IVA4* Pro LI GG

DC970 F1	ATGAAGACTTTACGGGTCTCAGCGGCTCTCTCTCGAACTCTTTCGTC	*CNGC.IVA5* Pro LI GG
DC971 R1	TAGAAGACAAGAGTCCTGTTAAGAAGTGGAGTTCG	*CNGC.IVA5* Pro LI GG
DC972 F2	TAGAAGACAAACTCAGTCACCTACTCTTTCTGT	*CNGC.IVA5* Pro LI GG
DC973 R2	ATGAAGACTTCAGAGGTCTCACAGAGCTTCCTCAATAATTCTGTAATTTT	*CNGC.IVA5* Pro LI GG
DC1034 F1	ATGAAGACTTTACGGGTCTCACACCATGGCTCACACTAGGACTTTCAC	*AtCNGC19* NT LI GG
DC1035 R1	TAGAAGACAAGAACACGAAATCATCTTCAGGTGG	*AtCNGC19* NT LI GG
DC1036 F2	TAGAAGACAAGTTCAAAAACGCAAATCTCTTGAG	*AtCNGC19* NT LI GG
DC1037 R	ATGAAGACTTCAGAGGTCTCACCTTGACTTGAACAAATTTGGAATGAGG	*AtCNGC19* NT LI GG
DC1038 F	ATGAAGACTTTACGGGTCTCACACCATGGCTTCCCACAACGAAAACG	*AtCNGC20* NT LI GG
DC1039 R	ATGAAGACTTCAGAGGTCTCACCTTGGTTTGAACCTCTTTGGCATGA	*AtCNGC20* NT LI GG
