# Supplementary material for: A quantitative hypermorphic CNGC allele confers ectopic calcium flux and impairs cellular development
Source: eLife. 2017 Sep 21;6:e25012. doi: 10.7554/eLife.25012 (PMC5716663; doi:10.7554/eLife.25012)
Supplement: Supplementary file 2. [file elife-25012-supp2.pdf]

**Supplementary File 2. DNA plasmids used in this study.**

| ID | Complementation | Backbone | Selection Marker | Cloning Method |
|---|---|---|---|---|
| DC001 | $BRUSH_{pro}:BRUSH_{genomic}$ | pK7RWG2 (-35S) | *Kanamycin* | Gateway + Digest |
| DC002 | $UBQ_{pro}:BRUSH_{genomic}$ | pUB-GW-GFP | *35S:GFP* | Gateway |
| DC003 | $UBQ_{pro}:brush_{genomic}$ | pUB-GW-GFP | *35S:GFP* | Gateway |
| DC004 | $UBQ_{pro}:CNGC.IVA3_{CDS}$ | pUB-GW-GFP | *35S:GFP* | Gateway |
| DC005 | $UBQ_{pro}:CNGC.IVA4_{CDS}$ | pUB-GW-GFP | *35S:GFP* | Gateway |
| DC006 | $UBQ_{pro}:CNGC.IVA5_{CDS}$ | pUB-GW-GFP | *35S:GFP* | Gateway |
| DC007 | $UBQ_{pro}:AtCNGC19_{CDS}$ | pUB-GW-GFP | *35S:GFP* | Gateway |
| DC008 | $UBQ_{pro}:AtCNGC20_{CDS}$ | pUB-GW-GFP | *35S:GFP* | Gateway |
| | **RNAi** | **Backbone** | **Selection Marker** | **Cloning** |
| GC287 | $UBQ_{pro}$*:BRUSH 5’RNAi* | BB53 LIIIβ A-B | *UBQ10:NLS-2xYFP* | Golden Gate |
| GC288 | $UBQ_{pro}$*:BRUSH 3’RNAi* | BB53 LIIIβ A-B | *UBQ10:NLS-2xYFP* | Golden Gate |
| | **Promoter:GUS** | **Backbone** | **Selection Marker** | **Cloning** |
| GC219 | $BRUSH_{pro}:GUS$ | BB53 LIIIβ A-B | *UBQ10:GFP* | Golden Gate |
| GC320 | $CNGC.IVA3_{pro}:GUS$ | BB53 LIIIβ A-B | *UBQ10:NLS-2xYFP* | Golden Gate |
| GC321 | $CNGC.IVA4_{pro}:GUS$ | BB53 LIIIβ A-B | *UBQ10:NLS-2xYFP* | Golden Gate |
| GC322 | $CNGC.IVA5_{pro}:GUS$ | BB53 LIIIβ A-B | *UBQ10:NLS-2xYFP* | Golden Gate |
| | ***Xenopus laevis*** | **Backbone** | **Selection Marker** | **Cloning** |
| GC188 | $T7_{pro}$*: brush* | pSEVA191 1-2 | Carbenicillin | Golden Gate |
| GC189 | $T7_{pro}$*: BRUSH* | pSEVA191 1-2 | Carbenicillin | Golden Gate |
| GC190 | $T7_{pro}$*: brush -YFP* | pSEVA191 1-2 | Carbenicillin | Golden Gate |
| GC191 | $T7_{pro}$*: BRUSH -YFP* | pSEVA191 1-2 | Carbenicillin | Golden Gate |
| GS40 | $T7_{pro}$*: VN-BRUSH* | pSEVA191 1-2 | Carbenicillin | Golden Gate |
| GC313 | $T7_{pro}$*: VC-BRUSH* | pSEVA191 1-2 | Carbenicillin | Golden Gate |
| GC262 | $T7_{pro}$*: BRUSH –VC* | pSEVA191 1-2 | Carbenicillin | Golden Gate |
| GS39 | $T7_{pro}$*: VN-brush* | pSEVA191 1-2 | Carbenicillin | Golden Gate |
| GS41 | $T7_{pro}$*: VC- brush* | pSEVA191 1-2 | Carbenicillin | Golden Gate |
| GC261 | $T7_{pro}$*: brush –VC* | pSEVA191 1-2 | Carbenicillin | Golden Gate |
| GC609 | $T7_{pro}$*: KAT1 –VC* | pSEVA191 1-2 | Carbenicillin | Golden Gate |
| GC610 | $T7_{pro}$*: KAT1 –VN* | pSEVA191 1-2 | Carbenicillin | Golden Gate |
| | **Yeast Two-Hybrid** | **Backbone** | **Selection Marker** | **Cloning** |
| DC009 | BD-BRUSH NT | pGBKT7-GW | Tryptophan | Gateway |
| DC010 | AD-BRUSH NT | pGADT7-GW | Leucine | Gateway |
| DC011 | AD-brush NT | pGADT7-GW | Leucine | Gateway |
| DC012 | AD-CNGC.IVA3 NT | pGADT7-GW | Leucine | Gateway |
| DC013 | AD-CNGC.IVA4 NT | pGADT7-GW | Leucine | Gateway |
| DC014 | AD-CNGC.IVA5 NT | pGADT7-GW | Leucine | Gateway |
| | **Split Ubiquitin** | **Backbone** | **Selection Marker** | **Cloning** |
| DC015 | BRUSH-Cub | pMetYC_GW | Leucine | Gateway |
| DC016 | NubWT-BRUSH | pNWTX_GW | Tryptophan | Gateway |
| DC017 | BRUSH-NubWT | pXNWT_GW | Tryptophan | Gateway |

| | | | | |
|---|---|---|---|---|
| DC018 | Positive Control | pNubWT-X_Gate | Tryptophan | Gateway |
| DC019 | Negative Control | pNX_Gate32-3HA | Tryptophan | Gateway |
