# Supplementary material for: A quantitative hypermorphic CNGC allele confers ectopic calcium flux and impairs cellular development
Source: eLife. 2017 Sep 21;6:e25012. doi: 10.7554/eLife.25012 (PMC5716663; doi:10.7554/eLife.25012)
Supplement: Supplementary file 3. [file elife-25012-supp3.pdf]

Supplementary File 3. List of key plant material used in this study (internal Zopra database IDs)

Seed ID	Plant No.	Description	Generation	Comment
2601	SL0979	Mutant Seed	M1	
10978	SL0979-2	Mutant Seed	M2	
6125	H474	Mutant Seed	M3	
8656	J6899	Mutant Seed	M4	
8656	J6900	Mutant Seed	M4	
8656	J6901	Mutant Seed	M4	
8656	J6902	Mutant Seed	M4	
55896		J6900 M4 x MG-20	F1	
55897		J6900 M4 x MG-20	F1	
55898		J6900 M4 x MG-20	F1	
55899		J6900 M4 x MG-20	F1	
55900		J6900 M4 x MG-20	F1	
55901		J6902 M4 x MG-20	F1	
59952		Mutant Seed	M5	
59953		Mutant Seed	M5	
59954		Mutant Seed	M5	
	L8257	Mutant	M5	Next-Gen Sequenced
	L2704	Recombinant	F2	F3 Mutant
	L2717	Recombinant	F2	F3 Segregating
	L5936	Recombinant	F2	F3 Segregating
	L6492	Recombinant	F2	F3 Segregating
	L6499	Recombinant	F2	F3 Segregating
	L6500	Recombinant	F2	F3 Segregating
	L6920	Recombinant	F2	F3 Segregating
11483	SL1484	Mutant Seed	M2	
31484	SL1484-1	Mutant Seed	M3	
87136	SL1484-1	Mutant Seed	M4	
